# Disease mentions in airport and hospital geolocations expose dominance of news events for disease concerns

**DOI:** 10.1186/s13326-018-0186-9

**Published:** 2018-06-12

**Authors:** Joana M. Barros, Jim Duggan, Dietrich Rebholz-Schuhmann

**Affiliations:** 1 Insight Centre for Data Analytics, Data Science Institute, NUI Galway, Lower Dangan, Galway, Ireland; 2 School of Computer Science, NUI Galway, University Road, Galway, Ireland; 3 ZB MED, University Cologne, Gleueler Str. 60, Cologne, 50931 Germany

**Keywords:** Social media, Disease surveillance, SNOMED-CT, MetaMap, Part-of-speech tagging, Geolocation

## Abstract

**Background:**

In recent years, Twitter has been applied to monitor diseases through its facility to monitor users’ comments and concerns in real-time. The analysis of tweets for disease mentions should reflect not only user specific concerns but also disease outbreaks. This requires the use of standard terminological resources and can be focused on selected geographic locations. In our study, we differentiate between hospital and airport locations to better distinguish disease outbreaks from background mentions of disease concerns.

**Results:**

Our analysis covers all geolocated tweets over a 6 months time period, uses SNOMED-CT as a standard medical terminology, and explores language patterns (as well as MetaMap) to identify mentions of diseases in reference to the geolocation of tweets. Contrary to our expectation, hospital and airport geolocations are not suitable to collect significant portions of tweets concerned with disease outcomes. Overall, geolocated tweets exposed a large number of messages commenting on disease-related news articles. Furthermore, the geolocated messages exposed an over-representation of non-communicable diseases in contrast to infectious diseases.

**Conclusions:**

Our findings suggest that disease mentions on Twitter not only serve the purpose to share personal statements but also to share concerns about news articles. In particular, our assumption about the relevance of hospital and airport geolocations for an increased frequency of diseases mentions has not been met. To further address the linguistic cues, we propose the study of health forums to understand how a change in medium affects the language applied by the users. Finally, our research on the language use may provide essential clues to distinguish complementary trends in the use of language in Twitter when analysing health-related topics.

## Background

The increase in life expectancy through better health of the world population has mainly been achieved through advancements in the fields of medicine, biology and microbiology [1]. However, it becomes increasingly crucial to public health research to detect, monitor, treat and avoid threats to population health [2]. Thus, public health has benefited from the use of surveillance [3] which has been crucial for the detection of disease outbreaks and its counter-actions in our modern information society. This has become a key issue for public health and has led to the application of new sources of valuable health information.

Modern sources of data such as search engine queries [4] and online news [5, 6] can provide near real-time, government independent information through different channels, and have been harnessed in the health domain. In recent years, social media networks have moved into the focus of the research; this medium fosters the sharing of health-related content (e.g. personal experiences and opinions), thus, being the preferred platform for sharing information [7]. One of such platforms is Twitter [8]. This resource is being used by over 310 million users worldwide [9] who publish their messages to the public (i.e. tweets) possibly in combination with the location of the individual; thus, it supplies a continuous stream of data useful to monitor public health concerns such as disease spread. Twitter has been exploited to monitor disease awareness and surveillance [10–13], suggesting its usefulness for evaluating the health state of a population. The available location information helped to identify global movement patterns [14] and has been integrated into specific applications in the health domain [15–17].

Given the richness of this source, we take advantage of the rapid availability of data, textual features and geolocation provided from Twitter. We focus on the full range of illnesses, including infectious and non-communicable diseases, to determine the scope of disease mentions in social media. The origin of the tweets is given special attention to contrast hospital geolocated tweets with those from airports. Furthermore, we address the linguistic cues, provided by the users, when health is discussed. By comparing both infectious and non-infectious diseases, we hope to discover if language and/or locations features can be used to uniquely characterise these categories. This research is based on the hypothesis that large-scale social media data can provide new insights about the health state of the population through the analysis of language and with a focus on location.

Our research is based on the following assumptions:

1. Twitter is a prime news medium where a wide range of illnesses are discussed. This enables the detection of different patterns in the discussion of selected diseases, and as a consequence allows linking of worldwide events with such disease mentions.
2. Considering the location plays an important role in determining the relevant health mentions and in monitoring specific areas for their distribution of health mentions. As a primary assumption, we expected that a hospital location would inflate the number of disease mentions, given the purpose of the location.
3. Different language styles could be predominant when communicating different illnesses; knowing the language patterns could help to identify non-explicit mentions of a given disease. Furthermore, different language patterns may be attributed to different locations.

## Related work

In the health domain, there is an increased interest in the use of social media analytics. The first exploitation of Twitter in this regard was performed by [18] to improve market predictions based on external information, in this case, using the public belief concerning the likelihood that H1N1 (i.e. swine flu) would turn into a pandemic. For the case of specific diseases and with the focus on the health state of the population, Twitter was initially tested for the case of influenza (i.e. flu) in the areas of surveillance and prediction [12, 19–21]. This illness was comprehensively researched due to the availability of well documented and historic gold standard data, its seasonality, and its ease in infecting others [22]. In this case study, more attention was initially given to specific words (i.e. keywords) or individual words (i.e. unigrams) present in a tweet to select potentially relevant messages [12, 19–21]. However, further developments led to machine learning approaches which take advantage of additional features such as n-grams [11, 23], regular expressions [13], user behaviour [24], and part of speech [25] to further filter relevant messages. With the results achieved for influenza, other diseases such as Ebola [26], food-borne illnesses [27], respiratory illnesses [28], and mental health diseases [29], were researched following comparable methodologies. Geolocation has been harnessed to focus on specific cities, regions, and countries [12, 17, 30, 31] and to study disease diffusion networks [15, 16, 23, 27].

Given this, there is still research to be conducted regarding how the proximity to disease-prone locations influences Twitter users, especially at a language level. In addition, these findings could elucidate on what and how information is shared. There is also a lack of research regarding the identification of multiple diseases from tweets, although, this is partially addressed by topic modelling approaches [32, 33].

Following the same principles described above, the analysis of language could be used to distinguish non-communicable disease mentions in tweets from infectious disease mentions. For example, it is expected that users apply a different language when being concerned about cancer and food poisoning. So far, Twitter has been used to analyse specific disease outbreaks, which required to capture specific mentions of disease. By contrast, we observe in this study the full spectrum of disease terms to better analyse the language use of disease mentions on Twitter for the outbreak or trend development of different disease types.

Furthermore, we use the geolocation to differentiate Twitter use for hospital visitors in contrast to airports. In our primary assumption, we expected that Twitter use in hospitals is focused on specific disease mentions, whereas the use of Twitter at airports could form an indicator for the early detection of communicable diseases. Certainly, the geolocation restricts the amount of Twitter data and the use of medical terminology for the identification of disease mentions may not necessarily reflect the mention of diseases in the daily common language in the use of Twitter. However, monitoring the full amount of data published through Twitter should give sufficient input to analyse the questions addressed above.

## Methods

### Data

The collected Twitter data amounts to 58’751’297 tweets gathered between the 26th of October 2016 and the 27th of March 2017. This was performed using the Twitter application program interface (API) [34] by collecting only tweets containing latitude and longitude coordinates (i.e. geolocated tweets) and written in English, using the API language filter. These messages were stored using MongoDB [35] due to its document-oriented construction, efficiency in querying large quantities of documents and scalability [36]. To improve the signal-to-noise ratio, we applied regular expressions to remove job advertisements (e.g.“We’re #hiring! Read about our latest #job opening here: St. Louis Trauma Hospital Seeking Multiple Specialties”) and predefined location sharing messages (e.g.“I’m at Terrabela Zona Sul in Porto Alegre, RS”). Subsequently, the data set was filtered according to the proximity of tweets to airports (airport collection) and hospitals (hospital collection). We chose an area within a 3 km distance surrounding the airports, motivated by the large size of an airport, and a 0.2 km radius surrounding hospitals. The remaining set of messages constitutes the geolocated collection. The airport coordinates were retrieved from the OurAirports [37] database; after considering only large airports the result consisted of 575 locations. Regarding the hospitals, to gather a large sample we utilise Open Street Map [38] to automatically collect 77’989 locations worldwide.

With this partition of the data, we can focus on identifying the differences that location poses on the frequency and language for distinct illnesses. In particular, the targeted locations that constitute “disease hot spots” due to their nature. Increases in international travel are raising concerns regarding travel-associated illnesses [39], and hospitals are inherently prone to have a high frequency of sick people.

### Disease terms

**Fig. 1:**
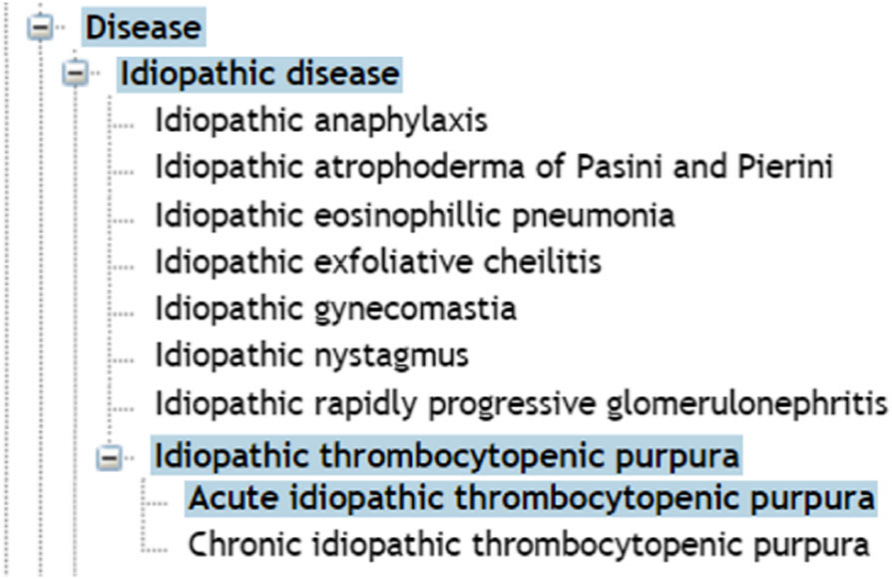
Disease terms retrieval. The disease terms were collected from the Disease class and its sub-classes. In the example “acute idiopathic thrombocytopenic purpura” is a sub-class of “idiopathic thrombocytopenic purpura”, and this disease is a sub-class of “idiopathic disease”

Clinical terms have been collected from the Systematized Nomenclature of Medicine – Clinical Terms [40], the reference source for a comprehensive and precise coverage of clinical terms. These terms, referred to as “disease terms” in this manuscript, have been selected from the Disease class of the terminology, as depicted in Fig. 1. The retrieval was performed through the Bioportal’s API [41] and, to achieve a broader scope, for each class the corresponding sub-classes and synonyms have been collected. The synonyms enable the normalisation of the diseases names, i.e.layman’s terms are considered as well as proper medical terminology. Due to the limits in Twitter’s message length (in characters) and due to the complexity of specific disease terms, we decided to remove names which are composed of more than three terms without considering determiners, conjunctions and prepositions (i.e. “of”, “from”, “the”, “a”, “and”, “to”), which were frequently observed in the list of disease terms. The remaining disease terms and synonyms were utilised for the selection of relevant tweets. The search for the retrieved terms and synonyms was performed using the complete set of terms (e.g. “muscle atrophy” was searched as a strict term in contrast to combinatorial variants of “muscle” and “atrophy”), followed by a search using the synonym list.

### Part-of-speech tagging

**Fig. 2:**
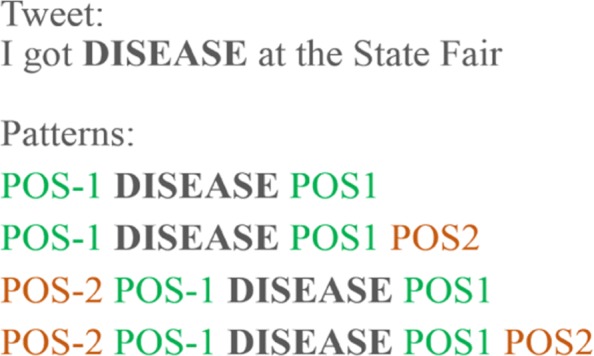
POS patterns example. Patterns are created around the disease term. When it is not possible to have an equal number of POS tags on each side, the pattern stops. For this example, four patterns were created

Tweets contain a variety of special characters, therefore, we applied five modifications to increase the performance of the pattern identification: (1) the disease term, if present, was normalised into “DISEASE”; (2) usernames, defined by words immediately preceded by the symbol @, were replaced by “@username”; (3) URLs were replaced by “URL”; (4) the @ symbol was replaced by “at” when not succeeded or preceded by a word; and (5) the remaining punctuation symbols have been removed. Subsequently, each tweet was tokenized and a Part-of-Speech (POS) tag was assigned to each token. These steps were performed using the TweetTokenizer and the POS tagger from Python’s Natural Language Tool Kit (NLTK) package [42], as well as the Penn Treebank tag set for the POS tagger. It was decided to focus on POS due to their ability to provide a general grammatical tag based on a word definition and its context. NLTK was chosen given its widespread use and good performance. To produce the POS patterns, a rule-based approach, exemplified in Fig. 2, was followed. This approach permitted to focus on POS tags surrounding the disease term which we hypothesise being related to the semantics of the disease term in the tweet.

### Named entity recognition

The disease terminology collected for this research includes polysemous terms mainly due to the presence of synonyms for some of the illnesses. Although the consideration of synonyms allows for a more appropriate representation of the layman language used on Twitter, it can also lead to the dubious semantics of the terms, rendering the tweet unusable for monitoring the health status of the population sample. To address this issue, we apply Named Entity Recognition (NER) techniques. With this, we try to classify entities (i.e. disease names is this case) contained in each tweet. Given that disease terms are not common entities in NER tools provided by popular services such as Stanford NER [43] and GATE’s TwitIE [44], we decided to use MetaMap [45]. MetaMap utilises the unified medical language system (UMLS) metathesaurus to identify concepts referenced in the presented text, the relevance is given through the MetaMap Indexing (MMI) score which has a maximum score of 1000 corresponding to highest relevance. Although specialised to biomedical text, we chose this tool given its suitability for the purposed task.

## Results and discussion

### Data exploration

The retrieval and filtering of the clinical terms amounted to 21’080 disease names and 19’813 synonyms. Both numbers differ due to the lack of synonyms for some disease terms.

The full analysis of all 58’751’297 with regards to disease mentions and geolocation produced the following result: 242 messages are within the 3 km radius of airports, 132 occur near hospitals, and the remaining 10’242 are assigned to the geolocated collection. From the 132 messages within the 0.2 radius from hospitals, 3 are simultaneous within 3 km of airports. In total, 10’613 potentially relevant tweets have been identified, i.e. containing a disease mention that could be normalised to the disease terms.

Given the contrast between the number of hospital locations and airport locations, the smaller number of retrieved messages occurring near hospitals suggest that: (1) the proximity to hospitals does not induce a significant number tweets covering disease mentions; and (2) there seems to be only a small number of users (with geolocation activated) tweeting near hospitals.

#### Data statistics

**Fig. 3:**
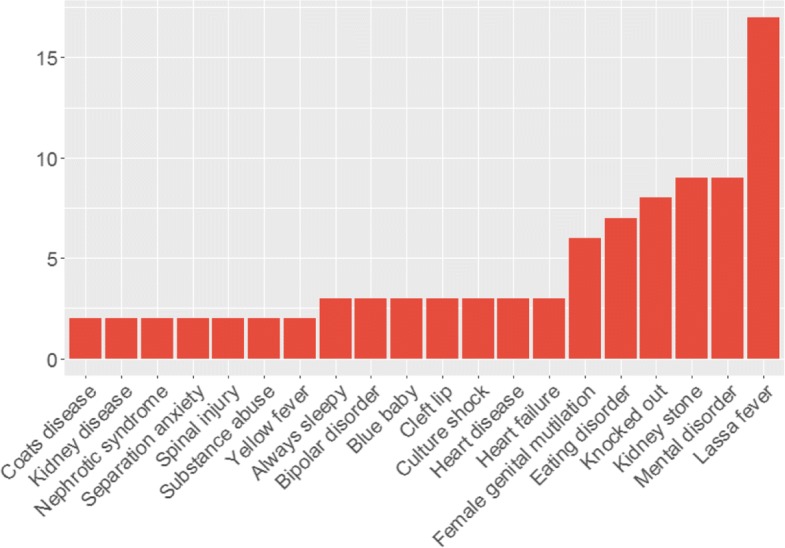
Terms distribution for the hospital collection. A total of 55 messages containing terms with a frequency of 1 were omitted for simplicity. In total, 61 disease terms are present in this collection

**Fig. 4:**
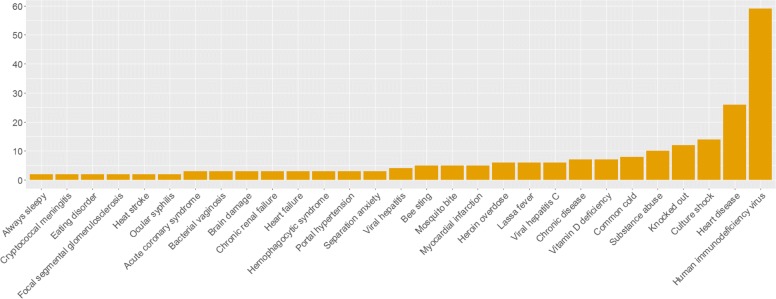
Terms distribution for the airport collection. Terms with a frequency of 1 (*n*=26) were omitted for simplicity. In total, 29 disease terms are present

**Fig. 5:**
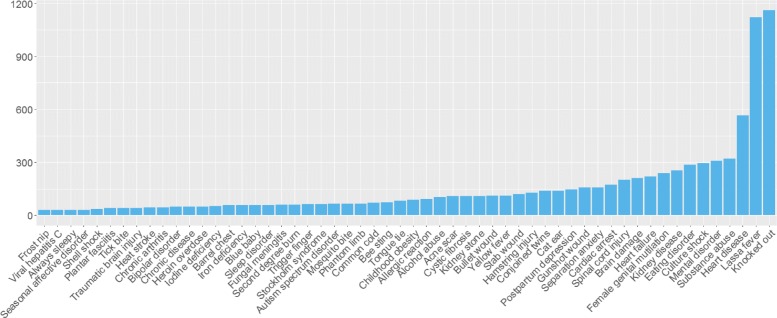
Terms distribution for the geolocated collection. Due to their high frequency and for simplicity, terms appearing in less than 30 messages were omitted (*n*=432). The total number of disease terms present is 56

Further analysing the complete set of 10’613 tweets, 493 disease terms and synonyms have been identified in the data set, with 302 present in more than 1 message. As a first step, we utilised tweets in each collection to determine the distribution of the disease terms. For the hospital and airport tweets, results are shown in Figs. 3 and 4 where terms with a frequency lower than 1 and 2, respectively, were omitted. The term distribution for the geolocated collection is present in Fig. 5. In this case, it was decided to only show the terms with a frequency higher or above 30. The decision to omit certain terms is due to simplicity and improved readability of the presented figures.

The results show that 21 disease terms are common to all collections, which include“viral hepatitis”, “always sleepy”, “heart failure”, “kidney stone”, “brain damage”, “mosquito bite”, among others. “Knocked out”, “Lassa fever”, “heart disease”, “substance abuse”, “mental disorder”, and “culture shock” are present in high frequency in both collections. The term ‘human immunodeficiency virus” (HIV), the most frequent in the airport collection, is absent in the geolocated and hospital collections. Upon further inspection, it was found that these messages were created by an organisation, which raises awareness of stigma, to share information about HIV related news.

Additionally, we compare the least frequent terms in all collections. For the geolocated sample, 383 terms appear in less than 10 messages. In the airport collection, 50 terms have a frequency below 10. The hospital collection has 55 terms with a frequency lower than 5. Considering the three samples, “multiple myeloma” is the only term common to all. Upon further inspection of the low frequency tweets, it was clear that personal statements were vastly more common than the presence of news titles, especially in the geolocated collection. For the airport and hospital collections this occurs to a lesser extent.

These results suggest that less mentioned terminology, in our dataset, is correlated with an increase in personal messages, which are useful to monitor the population’s health, and more frequent term occurrences are inflated due to the repetitiveness of news article titles. However, term specificity is another important factor. Disease terms such as “acute laryngopharyngitis”, “metabolic acidosis”, “segmental vitiligo”, and “plantar fasciitis” use specialised terminology of which the vast majority of the population is unaware. This may suggest the frequency of 1 for these terms. On contrary, terms such as “bee sting” and “mosquito bite”, although appearing in 5 messages, use informal terminology more characteristic of Twitter’s users.

For the second step, we consider all collections as a single dataset. For the remaining of the paper, we will focus on terms with an occurrence frequency above 199; this subset corresponds to ∼ 50% of the Twitter data collection hence we believe it to be appropriate for further analysis and it provides a reasonable amount of messages for each disease mention. Additionally, we utilised this threshold given the high volume our data and to guarantee readability throughout the paper. All the terms found correspond to the diseases’ clinical terminology, i.e. the terms are not synonyms. The filtering of terms with more than three words reduced the complexity hence their presence in the data set, given that we expect simple English to be preferentially used in Twitter. The disease terms range from clear health-related terms (e.g. “heart disease”, “mental disorder”, “brain damage”) to clinically less relevant terms (e.g. “knocked out”). All messages with a disease term have been further analysed to determine the distribution of terms and the use of language in the tweets. The results of this analysis are partly presented in Table 1.

**Table 1:** Disease terms message analysis

	Message content
Lassa fever	The term is only applied in the context of news-related messages.
Heart disease	The term is used to express health concerns in news reports, raise awareness and for personal statements.
Substance abuse	The messages contain news article titles, personal tweets and awareness tweets. These messages also include job advertisements which were not filtered by the previous steps.
Mental disorder	The term is used in personal messages and in tweets related to awareness.
Eating disorder	The messages are related to news stories and personal opinions.
Kidney disease	The term is mostly used in news stories, to a lesser extent it is applied in personal tweets.
Female genital mutilation	All messages correspond to news articles.
Heart failure	The majority of the messages correspond to news articles and job advertisements unfiltered by the previous steps. The remaining messages are personal statements from the users.
Brain damage	The messages include news stories and personal tweets in which the disease term is applied in a clinical sense. The remaining messages use a term with a non-clinical meaning.
Spinal cord injury	The term is mostly applied in the context of news-related messages and job advertisements. The remaining tweets correspond to personal statements.

Content analysis for “knocked out” and “culture shock” is present in text

Considering the disease terms “knocked out” and “culture shock”, the messages may use the terms in a non-clinical sense. The first term can be interpreted and often be used in the sense of elimination (e.g. “Novak Djokovic knocked out of Australian Open by 117th-ranked Denis Istomin to hand Andy Murray a huge boost URL.”), and “culture shock” is mostly used as a company name (e.g. “Cool new socks! Made in Chicago USA! at Culture Shock - Clothing and Records URL”). Similarly, “cat ear” clinically corresponds to a malformation of the inner ear [46]; however, in the tweets, the term is used to express a clothing item (“e.g. I don’t like Halloween I just like being able to wear cat ears again URL”). Similar results, although to a lesser extent, can be identified with “brain damage” and “cardiac arrest” which are used in song names (e.g. “Brain Damage by Pink Floyd is #nowplaying in Vera’s On The Drive, Vancouver.”, “#PalmillaBeach pool is #nowplaying Cardiac Arrest by #BadSuns #cubevenue”).

Our analysis and findings reveal that there is a strong presence and influence of news articles and their distribution on the use of medical language when properly analysing Twitter feeds. The majority of disease terms occur frequently in tweets with reference to specific news articles or explicitly repeat the article title. As a conclusion, news media are the source explicit disease term mentions and their frequency form a systematic bias to disease mentions and have to be excluded when analysing Twitter feeds for surveillance. This also shows that Twitter users give high relevance to news media; this phenomenon could receive particular relevance when determining the impact of campaigns (exposed as specific news events) that target given diseases (e.g. awareness campaigns). Furthermore, a short-term peak in the frequency of a disease term aligned with the increase in related news articles can be exploited as an indicator for changes in public concerns, perceptions, and opinions for an illness, or could be removed as an obvious distraction from the surveillance analysis.

#### Geographic distribution

**Fig. 6:**
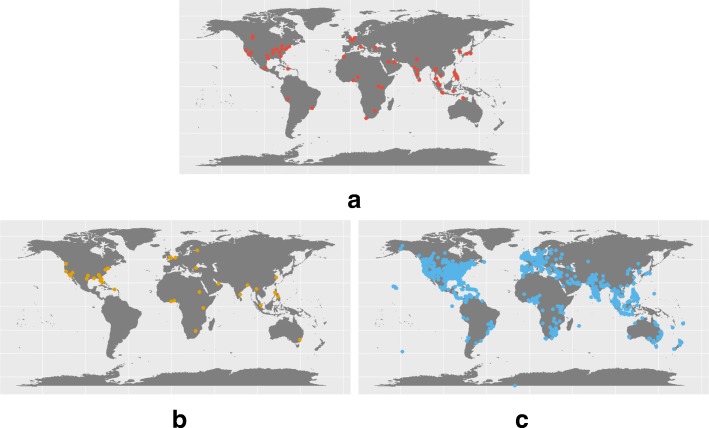
Geographic distribution for the dataset collections. **a** Hospital, **b** Airport, **c** Geolocated

**Fig. 7:**
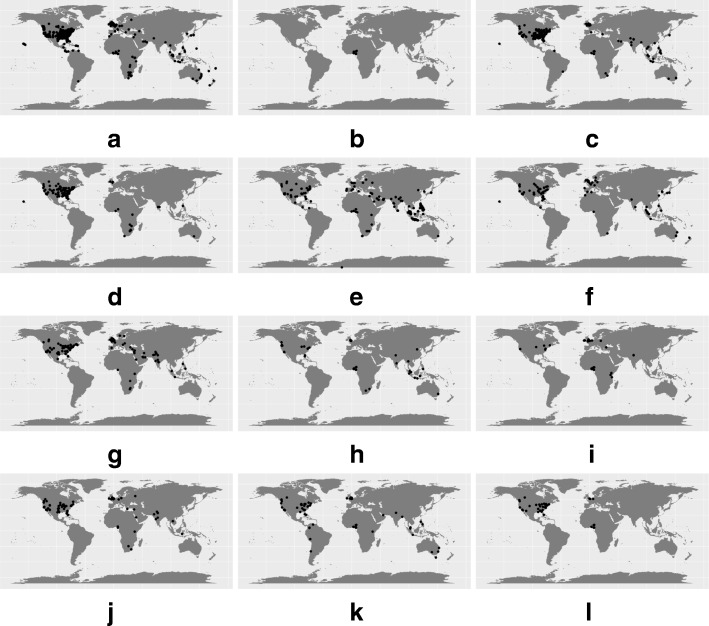
Disease terms’ geographic distribution. **a** Knocked out, **b** Lassa fever, **c** Heart Disease, **d** Substance abuse, **e** Mental Disorder, **f** Culture shock, **g** Eating disorder, **h** Kidney disease, **i** Female genital mutilation, **j** Heart failure, **k** Brain damage, **l** Spinal cord injury

For all 10’613 messages (see Fig. 6), we show the worldwide geographic distribution of the tweets assigned to each collection. The presence of English written tweets occurring in countries where English is not the native language occurs due to the high presence of news article titles written in English. With regards to the subset selected above, we present in Fig. 7 the geographic distribution of each disease term given the location of the messages. Overall, a large portion of tweets originates in the United States of America, since this country has the highest number of Twitter users [47].

As an exception, messages about “Lassa fever” originate from Nigeria and nearby regions, and are entirely constituted by news related tweets. We use this case to further explore the correlation between our Twitter dataset and other sources of health information, in an outbreak context. For this, we gathered past information from the World Health Organisation (WHO) website [48] and Google News [49] (using “Lassa fever” as a search term). Given the high presence of URLs in our Twitter dataset we hypothesise that news articles released at a given time-stamp could be the source of the high frequency of tweets. We then compared outbreak reports or news with spikes in the frequency of tweets mentioning “Lassa fever”. WHO provided scarce information, including the absence of reports from October 2016 to January 2017. In addition, we found no clear association between Google News and the news mentioned in the tweets. This can be due to the lack of sources from the major affected countries, in Google News. “Lassa fever” is considered endemic in regions of sub-developed countries where internet access is not widely available. However, this data can be leveraged to extract outbreak information from local news sources.

These findings suggest that attributing a location may reduce the capability of identifying disease outbreaks mainly relating to the high volume of news media content. In addition, the presence of news media hinders the content analysis of Twitter with the goal of monitoring the health state of a population.

### Part of speech

#### Disease term part of speech

Our POS analysis with regards to the disease mentions shows that the most frequent POS patterns are singular nouns (NN, 4’390 terms), and singular proper nouns (NNP, 4’201 terms). To a lesser degree, we identified adjectives (JJ, 350 terms), verbs in base form (VB, 339 terms), and prepositions or subordinating conjunctions (IN, 289 terms). For the majority of the disease terms, the preferential POS is an NNP or NN, a few exceptions are “knocked out” which is assigned an IN, as the second most frequent term, and “kidney disease” which is attributed a VB also as the second most frequent term. Due to the nature of the disease term, it was expected a high presence of nouns or proper nouns for selected diseases (e.g. “Lassa fever”), hence, the results suggest that the term could be used with its clinical meaning. Furthermore, in previous sections, we addressed the high presence of news articles which is a likely indicator that the term is used in a clinical context. However, it is applied to give reports regarding the disease and not to express personal statements from the users.

#### Part of speech patterns

Considering the POS patterns generated through the previously mentioned method, overall, the results suggest a strong presence of NNP. This predominance of nouns has already been addressed in the literature [50] and it was linked to the distinct vocabulary used on Twitter. These findings also suggest a relaxed use of regular grammar constructs such as the random capitalisation of words. In Tables 2, 3 and 4 we present a selection of the most frequent patterns for the hospital, airport and geolocated collections. In addition to the presence of NNP, the collections also contain IN and coordinating conjunctions (CC). Focusing on the patterns assigned to each disease, the strong presence of NNP is again present.

**Table 2:** POS patterns for the hospital collection

	Frequency
’DISEASE’,’NNP’	14
’IN’,’DISEASE’,’NNP’	6
’JJ’,’DISEASE’,’IN’	6

The table presents the top 3 patterns with a frequency higher than 5

**Table 3:** POS patterns for the airport collection

	Frequency
’NNP’,’DISEASE’,’NNP’	36
’NNP’,’NNP’,’DISEASE’,’NNP’	16
’NNP’,’DISEASE’,’NNP’,’NNP’	11
’IN’,’DISEASE’,’CC’	10

The table presents the top 4 patterns with a frequency higher than 9

**Table 4:** POS patterns for the geolocated collection

	Frequency
’IN’,’DISEASE’	592
’DISEASE’,’NNP’	589
’IN’,’DISEASE’,’NNP’	418
’NNP’,’DISEASE’,’NNP’	401

In this table it is represented the top 4 patterns with a frequency higher than 300

The frequency of each pattern is correlated with the number of messages related to news articles. These tweets contain the same news title, thus, the high frequency of given patterns in disease terms such as “Lassa fever” and “female genital mutilation”. A similar behaviour can be seen with “culture shock”, albeit, in this case, it is related to the standard pattern used for advertising the product of a company. In contrast, terms such as “substance abuse”, “brain damage” and “spinal cord injury” are present in patterns with similar frequencies. These are similarly linked to news articles, however, the titles and news stories contain more diversity and are also repetitive, therefore, contributing to the frequency of the patterns. In addition, the results also suggest that similar patterns, within each disease term, tend to occur with similar frequencies.

### Named entity recognition

MetaMap was able to identify disease terms in 3’489 tweets from the initial 10’618 messages containing the terminology. In the hospital collection, Metamap reached a score of 4.04 and identified terms in 44 messages from the total of 132 messages. A total of 45 terms such as “eating disorder”, “brain damage”, and “substance abuse” were not identified. Considering the airport collection, MetaMap achieved an average relevance score of 25.49 and was able to identify the terms contained in 183 messages out of 242 messages. A total of 23 terms were not identified, these include terms such as “bee sting”, “shell shock”, “Lassa fever” and “bipolar disorder”. In the geolocated collection, the average relevance score was 3.36 and MetaMap was able to identify the terms in 3’272 messages of a total of 10’242. Similarly to the airport and hospital collections, issues regarding the identification of terms in the tweets were verified. For example, despite representing the most frequent term “knocked out” was also the term most difficult to identify to MetaMap. The same occurs with “Lassa fever” and “culture shock”. Considering the agreement between MetaMap’s term identification and the actual terminology, the score is of 0.62 (the terms contained in 2’044 tweets are representative of the terminology present in the 3’272 messages in which MetaMap can identify a UMLS concept). As an example, we detail the specific case of the term “knocked out”. This term is present in 103 of the 3’272 tweets identified by MetaMap, however, it is never identified by MetaMap as “knocked out”; the algorithm identifies other disease terms in these 103 messages.

The results suggest a trade-off between the use of complex and colloquial terminology. Although more frequent in Twitter, health-related layman terms can pose significant challenges and require the application of domain-specific semantic disambiguation tools. In addition, we suspect the characteristics of the messages (e.g. short length and possible disregard for grammar rules) may have increased the difficulty for MetaMap’s algorithm. This is further supported by cases where the same terminology was found in some tweets and not in others, despite being referenced in both.

## Conclusion

In this paper, we tested an approach to determine the presence of linguistic patterns associated with diseases and we explored the representation value of a geolocated Twitter sample. We analysed the full body of Twitter feeds with geolocation over a period of 5 months. Using SNOMED clinical terms, we verified a higher presence of non-communicable diseases compared to infectious diseases. The division of the data showed that hospital and airport locations do not contribute to the increase in the number of disease mentions, contrary to our expectation.

We also identified questionable interpretations for selected disease terms, exposing non-medical interpretations of the medical term (e.g. “knocked out”, “culture shock”). The findings originating from our data exploration suggest a high presence of news article titles or mentions in the messages which indicate that current events have a strong influence on the disease’s frequency. An example is “Lassa fever”, the majority of the messages correspond to news stories originating from regions affected by an outbreak in late 2016/early 2017. This finding provides an alternative way to explore the news content provided by Twitter, mainly through the determination of the degree of concern of users and through the gathering of outbreak information from local news providers, thus, suggesting its utility for disease monitoring. However, our findings also suggest that Twitter is not only used as a medium to share personal statements but also to disseminate news articles. Furthermore, it suggests that users give high relevance and interest to news media.

Regarding the POS tagging, the majority tweets expose noun or proper noun use of the disease term which corresponds to our expectations and previous findings in the scientific literature. For the POS patterns, the high presence of news articles may have hindered the identification of linguistic patterns as the ones identified may solely relate to the news articles and not individual statements regarding health conditions. To address this, an additional step to remove tweets related to news articles could be implemented to exclusively analyse personal tweets. The results from the NER suggest that despite being useful to identify tweets containing the correct clinical terminology and providing semantic disambiguation, further developments are needed to better handle the unique style of the Twitter messages. To further address the linguistic cues, we propose the study of health forums to understand how a change in medium affects the language applied by the users. Furthermore, these insights can provide new information on the complexity of language when discussing health.

Using a collection of more than 58 million tweets, we used and determined language patterns, and contrasted the use of Twitter between airport locations (with a larger number of feeds) against the hospital location (with a small number of feeds). As an outcome, we determined that these locations are not suitable for the collection of significant portions of tweets concerned with disease outcomes. Additionally, we verified a high presence of discussion regarding non-communicable diseases.This study is based on the premise that users utilise Twitter as a medium to share concerns regarding illnesses and that hospital and airport locations would be preferential for the discussion of certain diseases or diseases in general. This was not verified, in contrast, we found the predominance and influence of news articles. To closely monitor disease outbreaks, personal statements mentioning illnesses or symptoms are desired. However, our findings can also be applied to measure the degree of concern expressed by the users, although not strictly indicative of an outbreak it can be used use to determine if additional public health measures should be implemented.
